# Sexual Development of the Hermaphroditic Scallop *Argopecten irradians* Revealed by Morphological, Endocrine and Molecular Analysis

**DOI:** 10.3389/fcell.2021.646754

**Published:** 2021-03-16

**Authors:** Huilan Wei, Wanru Li, Tian Liu, Yajuan Li, Liangjie Liu, Ya Shu, Lijing Zhang, Shi Wang, Qiang Xing, Lingling Zhang, Zhenmin Bao

**Affiliations:** ^1^MOE Key Laboratory of Marine Genetics and Breeding, Ocean University of China, Qingdao, China; ^2^Laboratory for Marine Biology and Biotechnology, Pilot National Laboratory for Marine Science and Technology (Qingdao), Qingdao, China; ^3^Laboratory of Tropical Marine Germplasm Resources and Breeding Engineering, Sanya Oceanographic Institution, Ocean University of China, Sanya, China; ^4^Laboratory for Marine Fisheries Science and Food Production Processes, Pilot National Laboratory for Marine Science and Technology (Qingdao), Qingdao, China

**Keywords:** sex differentiation, sex steroids, *Dmrt1L*, *FoxL2*, *Argopecten irradians*

## Abstract

Simultaneous or functional hermaphrodites possessing both ovary and testis at the same time are good materials for studying sexual development. However, previous research on sex determination and differentiation was mainly conducted in gonochoristic species and studies on simultaneous hermaphrodites are still limited. In this study, we conducted a combined morphological, endocrine and molecular study on the gonadal development of a hermaphroditic scallop *Argopecten irradians* aged 2–10 month old. Morphological analysis showed that sex differentiation occurred at 6 months of age. By examining the dynamic changes of progesterone, testosterone and estradiol, we found testosterone and estradiol were significantly different between the ovaries and testes almost throughout the whole process, suggesting the two hormones may be involved in scallop sex differentiation. In addition, we identified two critical sex-related genes FoxL2 and Dmrt1L, and investigated their spatiotemporal expression patterns. Results showed that *FoxL2* and *Dmrt1L* were female- and male-biased, respectively, and mainly localized in the germ cells and follicular cells, indicating their feasibility as molecular markers for early identification of sex. Further analysis on the changes of *FoxL2* and *Dmrt1L* expression in juveniles showed that significant sexual dimorphic expression of *FoxL2* occurred at 2 months of age, earlier than that of *Dmrt1L*. Moreover, *FoxL2* expression was significantly correlated with estradiol/testosterone ratio (E_2_/T). All these results indicated that molecular sex differentiation occurs earlier than morphological sex differentiation, and *FoxL2* may be a key driver that functions through regulating sex steroid hormones in the scallop. This study will deepen our understanding of the molecular mechanism underlying sex differentiation and development in spiralians.

## Introduction

Sexual reproduction has long been the focus of research in the life sciences, and extensive research has been carried out in various taxa, such as mammals (Sinclair et al., 1990; Warr and Greenfield, 2012; Andersen et al., 2017), birds (Smith et al., 2009; Wang et al., 2017), fishes (Sandra and Norma, 2003; Parzefall et al., 2008; Desjardins and Fernald, 2009), and insects (Hales et al., 2002; Erickson and Quintero, 2008). In many organisms, sex is genetically determined by the gene on the sex chromosome, which in turn activates downstream genes or pathways for sex differentiation and maintenance. For example, in mammals, sex is determined by the SRY gene on the Y chromosome, which activates SOX9 and inhibits female pathway. The decision in the gonad further specifies sex differentiation primarily by sex steroid hormones including androgens and estrogens (Matson and Zarkower, 2012).

Mollusca represents the second largest phylum of invertebrates after Arthropoda. It contains several subgroups, including cephalopods, gastropods, bivalves, monoplacophorans, etc. These animals exhibit a diversity of sexual systems, including gonochorism, simultaneous hermaphroditism, and sequential hermaphroditism (Collin, 2013). Specifically, cephalopods, scaphopods, and monoplacophora are mostly gonochoristic, solenogastres (a group of shell-less, worm-like aplacophoran mollusks) are simultaneous hermaphrodites, whereas bivalves and gastropods are most diverse, with gonochorism, simultaneous hermaphroditism, and sequential hermaphroditism (Collin, 2013; Breton et al., 2018). Therefore, there is a great potential for understanding the molecular mechanisms underlying gonochorism and hermaphroditism within bivalvia or gastropoda.

In mollusks, most studies on sex determination and differentiation have been conducted in gonochoristic and sequentially hermaphroditic organisms. For example, in the gonochoristic scallop *Patinopecten yessoensis*, FoxL2 and Dmrt1L have been identified as key sex genes and used for determining the timing of sex differentiation (Li et al., 2016, 2018). In another gonochoristic scallop *Chlamys farreri*, a sex-related quantitative trait locus has been reported (Jiao et al., 2014) and several key sex genes in vertebrates have been characterized, such as FoxL2 (Liu et al., 2012), Wnt4 (Li et al., 2013), and β-catenin (Li et al., 2014). In the sequential hermaphrodites, a potential sex-determining pathway containing SoxH, Dmrt, and FoxL2 has been proposed in the Pacific oyster *Crassostrea gigas* (Zhang et al., 2014), and Fem1-like, Dmrt, and FoxL2 are considered as key sex-determining genes in the pearl oyster *Pinctada margaritifera* (Teaniniuraitemoana et al., 2014, 2015). Recent studies reported the transcriptome sequencing of two simultaneous hermaphroditic bivalves, lion-paw scallop *Nodipecten subnodosus* and giant clam *Tridacna squamosa*, from which several sex-related genes were identified (Galindo-Torres et al., 2018; Li et al., 2020). But as yet, research on the sexual development of simultaneous hermaphroditic mollusks is still in its infancy.

The bay scallop *Argopecten irradians* is native to the east coast of the United States. It was introduced to China in the early 1980s, and has become a commercially important species widely cultured in northern China. As a simultaneous hermaphrodite, *A. irradians* has an independent gonad with distinct male (proximal) and female (distal) portions (Beninger and Le Pennec, 2016). It reaches sexual maturity at 1 year old, when the male and female gametes are released simultaneously. Therefore, *A. irradians* represents a good material for studying sex determination and differentiation. In present study, we collected scallops from 2 to 10 months of age, and investigated their gonadal development from the morphological, endocrine, and molecular standpoint. It will contribute to a comprehensive understanding on the mechanism underlying sexual development in simultaneous hermaphrodites.

## Materials and Methods

### Sample Collection

**FIGURE 1:**
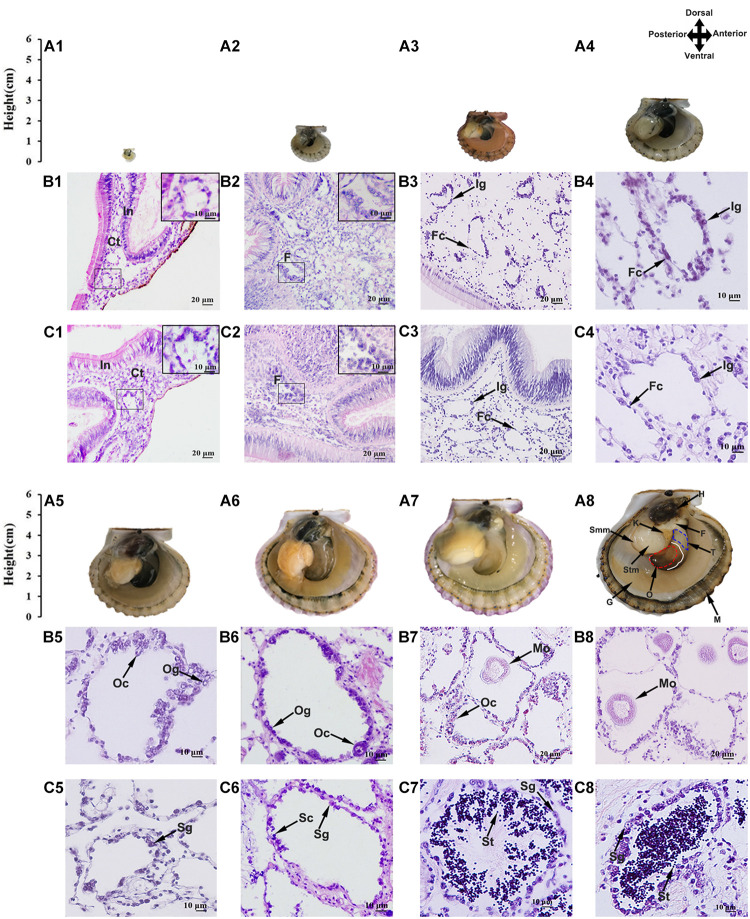
Morphological observation of juvenile scallops aged 2–10 months old. **(A)** Anatomy of the scallop showing the shell height and gonad morphology (the right shell valve was removed). Anterior toward right and posterior toward left. The gonad has distinct male and female portions, with the testis being proximal and the ovary distal. The solid white line indicates the boundary of ovary and testis, and the red and blue dotted lines mark the ovary and testis we collected, respectively. H, hepatopancreas; F, foot; T, testis; O, ovary; M, mantle; G, gill; Stm, striated muscle; Smm, smooth muscle. **(B)** Paraffin sections of the ovaries. **(C)** Paraffin sections of the testes. **(A1,B1,C1)** 2-month-old; **(A2,B2,C2)** 3-month old; **(A3,B3,C3)** 4-month old; **(A4,B4,C4)** 5-month old; **(A5,B5,C5)** 6-month old; **(A6,B6,C6)** 7-month old; **(A7,B7,C7)** 9-month old; **(A8,B8,C8)** 10-month old. In, intestine; Ct, connective tissues; F, follicle; Fc, follicle cell; Ig, indistinguishable gonium; Og, oogonium; Oc, oocyte; Mo, mature oocyte; Sg, spermatogonium; Sc, spermatocyte; St, spermatid.

Juvenile scallops used in this experiment were produced in the spring of 2017 and cultivated in Changdao, Yantai City, Shandong Province (38°17′43″N, 120°48′28″E). From May 2017 to January 2018, approximately 100–200 scallops were collected and sent back to the lab each month. The scallops were then acclimated in filtered and aerated seawater for 1 week at the temperature they were collected, varying from 4.6°C (in January) to 22°C (in August). About 40 healthy scallops were dissected each time, and their testes and ovaries were obtained. In order to discriminate testes and ovaries, only the portions at the ends of the gonad were collected (see the dotted lines in Figure 1). Parts of the gonads were immediately frozen in liquid nitrogen and stored at −80°C for hormone and RNA extraction. The other parts were fixed in 4% paraformaldehyde overnight, dehydrated with serial methanol (25, 50, 75, and 100%) diluted in 0.01 M phosphate-buffered saline and then stored at −20°C for paraffin sectioning.

For the 10-month-old scallops, different tissues including the mantle, gill, foot, ovary, testis, kidney, hepatopancreas, smooth muscle and striated muscle were obtained, immediately frozen in liquid nitrogen and stored at −80°C for RNA extraction. Parts of the gonads were fixed and dehydrated for *in situ* hybridization.

### Histology

The samples were dehydrated by ethanol, cleared in xylene, embedded in paraffin wax, and cut into 5 μm-thick sections on a rotary microtome (Leica, Wetzlar, Germany). Serial sections were tiled on glass slides, dewaxed with xylene, hydrated with graded ethanol to water, and stained with hematoxylin. Subsequently, the sections were counterstained with eosin, dehydrated with ethanol, cleared with xylene, mounted with neutral balsam, and covered with coverslips. Finally, the sections were observed under a Nikon’s Eclipse E600 research microscope.

### Sex Steroid Analysis

To extract sex steroids, 0.1 g gonad was homogenized in 500 μL ice-cold H_2_O for 1 min, and sonicated for 3 min at 200 W. Then, 400 μL of 25 mM HCl was added and the mixture was incubated at 40°C for 15 min. After the addition of 1.25 mL 0.07 M Na_2_HPO_4_ (pH 7.4), the homogenates were extracted twice with 14 mL dichloromethane. The organic extracts were evaporated to dryness under nitrogen stream at room temperature, and the resulting pellet was dissolved in 500 μL EIA Buffer in the enzyme-linked immunosorbent assay (ELISA) kits (Cayman Chemical Company, United States). Progesterone, testosterone and estradiol concentrations were measured according to the manuals. To evaluate the recovery of our extraction method, a known amount of progesterone, testosterone and estradiol standards was incorporated into a pre-extracted homogenate. The recoveries of progesterone, testosterone, and estradiol were 93.77% (*N* = 4), 95.64% (*N* = 4), and 91.15% (*N* = 4), respectively. Steroid concentrations were determined using the equation obtained from the standard curves.

### Gene Identification and Phylogenetic Analyses

To identify the scallop FoxL2 and Dmrt1L, homologous protein sequences from other organisms were collected from NCBI and used as queries against the scallop gonadal transcriptomes by TBLASTN with an *e*-value threshold of 1e–5. The organisms include *Homo sapiens*, *Mus musculus*, *Gallus gallus*, *Xenopus laevis*, *Danio rerio*, *Dicentrarchus labrax*, *Oreochromis niloticus*, *Oncorhynchus mykiss*, *Crassostrea gigas*, *Lottia gigantea*, *Crassostrea virginica*, *Pinctada maxima*, *Pinctada martensii*, *Pinctada fucata*, *P. margaritifera*, *P. yessoensis*, *C. farreri*, *Mimachlamys nobilis*, *Lingula anatinas*, *Bombyx mori*, *Drosophila melanogaster*, *Caenorhabditis elegans*, and *Suberites domuncula*. The conserved domains of the proteins were predicted using SMART^1^. Multiple sequence alignments were performed by ClustalW2. The neighbor-joining trees were constructed using MEGA X software (Kumar et al., 2018). Bootstrapping with 1,000 replications was conducted to evaluate the phylogenetic tree.

### RT-qPCR

To determine the relative expression of *FoxL2* and *Dmrt1L*, nine tissues of 10-month-old scallops and gonads of 2–10 month-old scallops were used for RNA extraction. Total RNA was isolated using the conventional guanidinium isothiocyanate method and digested with DNase I (TaKaRa, Shiga, Japan) to remove potential DNA contamination. RNA qualities were assessed by agarose gel electrophoresis and spectrophotometry. First-strand cDNA was synthesized from 2 μg total RNA using oligo(dT)_18_ and MMLV reverse transcriptase (TaKaRa, Shiga, Japan) in a volume of 20 μl. The primers used in the qPCR were listed in Table 1 and elongation factor 1-alpha (EF1A) was used as an endogenous control for the normalization of gene expression (Teaniniuraitemoana et al., 2015; Li et al., 2016, 2019). For each group, 4 to 8 samples were assayed and all reactions were conducted in triplicate. To ensure that the RT-qPCR ran properly, no-template and no-reverse transcription controls, as well as positive controls were included in each run. The PCR was conducted using Light Cycler 480 SYBR Green I Master on a Light Cycler 480 Real-time PCR System (Roche, Mannheim, Germany) using the following program: 94°C for 10 min, followed by 40 cycles of 94°C for 15 s and 60°C for 1 min. To verify that a single product was amplified, melting curve analysis was performed for each reaction. The relative expression level of each gene was quantified using the 2^–ΔΔ^ ^Ct^ method.

**TABLE 1:** Sequences of primers used for RT-qPCR and *in situ* hybridization.

Gene name	Experiment	Primer sequences (5′–3′)
FoxL2	RT-qPCR	F: CGGAGGCTTTTACTGACACTATCG R: TCTCGCTGCTACACCGTATGAAACT
	*In situ* hybridization	F: AGCTAGCACCCCCTATCCCAGT R: TTAGTGTCTTCCTCAAACTTCCTGC F7: **TAATACGACTCACTATAGGG**AGC TAGCACCCCCTATCCCAGT R7: **TAATACGACTCACTATAGGG**TTA GTGTCTTCCTCAAACTTCCTGC
Dmrt1L	RT-qPCR	F: GTGACGGAGACACTCAGAAAGCCAT R: GTGTCATTCCTGCCTTAGGTTCG
	*In situ* hybridization	F: AGGATATGGGTCTGGTGGCG R: CCGGGAGGTCTGGAAGTTTT F7: **TAATACGACTCACTATAGGG**AGG ATATGGGTCTGGTGGCG R7: **TAATACGACTCACTATAGGG**CCGGG AGGTCTGGAAGTTTT
EF1A	RT-qPCR	F: ACTGGAACCTCCCAAGCCGAT R: TTTACACCAAGCGTGTAGGCGAG

**The bold characters in primers F7 and R7 indicate T7 promoter sequence.**

### *In situ* Hybridization

Based on the full-length cDNA sequence of *FoxL2* and *Dmrt1L*, 400–700 bp fragments were amplified using the gene-specific primers F and R (Table 1). The PCR products were cloned and sequenced to confirm their identity. Then, PCR was conducted using the F7 and R (for the sense probe) or F and R7 (for the anti-sense probe) primer pairs to generate templates for *in vitro* transcription. Digoxigenin-labeled sense and anti-sense probes were synthesized with a DIG RNA Labeling Kit (SP6/T7) (Roche, Mannheim, Germany). Approximately 5 μm sections were prepared and affixed to the slides with 0.1 mg/ml polylysine for 10 h at 37°C. The sections were rehydrated through a descending series of ethanol solutions and phosphate buffered saline with 0.1% Tween-20 (PBST). Then, the samples were digested at 37°C for 30 min using 2 μg/ml protease K, and prehybridized at 60°C for 6 h in hybridization solution (50% formamide, 5 × SSC, 5 mM EDTA, 1.5% blocking reagent, 0.1% Tween-20, 100 μg/ml ribonucleic acid). After hybridization with the RNA probes at 60°C for 16 h, the samples were washed six times at 60°C for 15 min and twice at room temperature for 10 min in maleic acid buffer (0.1 M maleic acid, 0.15 M NaCl, 0.1% Tween-20, pH 7.5). Finally, the sections were incubated with alkaline phosphatase-conjugated anti-digoxigenin antibody (Roche, Mannheim, Germany), and the signals were detected using NBT/BCIP.

### Statistical Analysis

Differences of sex steroid contents or gene expression levels between testes and ovaries from the same month were analyzed using paired-sample *t*-tests. One-way ANOVA followed by Duncan’s test was performed to compare the differences of each hormone contents among months within ovary or testis, and the differences of gene expression among tissues. *P*-values lower than 0.05 were considered statistically significant. All data were analyzed using SPSS 22.0.

## Results

### Histological Analysis of the Gonads

Considering that the bay scallops reach sexual maturation at 1 year old, we investigated the gonadal developmental procedures using juveniles aged 2 (shell height ∼2.9 mm) to 10 months old (shell height ∼65.0 mm) (Figure 1A). The gonads were firstly examined histologically (Figures 1B,C), and the cell types and developmental stages were determined following previous studies (Beninger and Le Pennec, 2016; Yu et al., 2017). As seen, the 2-month-old gonads contain a small portion of connective tissues, which are in close contact with intestines (Figures 1B1,C1). In the 3-month-old gonads, some follicles can be observed (Figures 1B2,C2). In the 4- and 5-month-old gonads, the follicles grew bigger, with a monolayer of follicle cells and sexually indistinguishable gonia surrounding the inner wall (Figures 1B3–C4). The 6-month-old gonads started to differentiate, and the 7-month-old gonads were going through proliferative stage, with an increasing number of cells along the follicular wall and the appearance of a small number of oocytes and spermatocytes (Figures 1B5–C6). The 9- and 10-month-old scallops were at the growing stage (Figures 1B7–C8), with various germ cells being observed in the follicles, including oogonia and oocytes in the ovary, and spermatogonia, spermatocytes and spermatids in the testis.

### Sex Steroid Analysis

**FIGURE 2:**
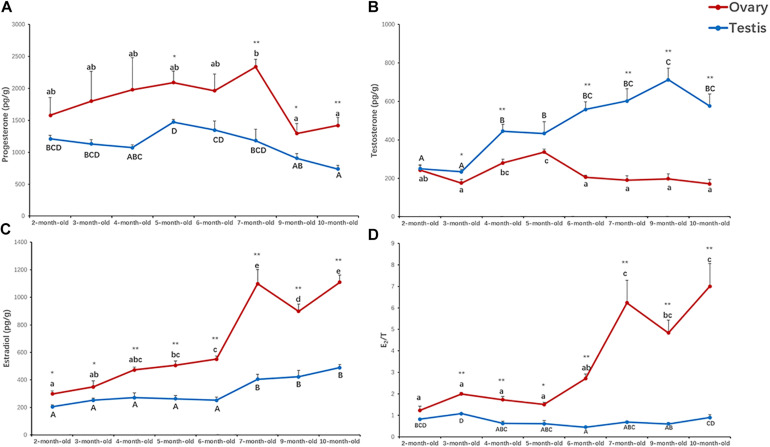
Concentrations of progesterone **(A)**, testosterone **(B)**, estradiol **(C)**, and estradiol/testosterone (E_2_/T) ratio **(D)** in the ovaries and testes of 2- to 10-month-old scallops. The vertical bars represent the means ± S.E. (*N* = 5). “*” indicates significant difference (*P < 0.05; **P < 0.01) between the ovaries and testes from the same month. Different letters indicate significant differences (*P* < 0.01) among months in the testes (big letters) or ovaries (little letters), i.e., letters shared in common among groups indicate no significant difference.

To evaluate the potential effects of sex steroids on gonadal development, we measured the concentrations of progesterone, testosterone and estradiol in the ovaries and testes of 2- to 10-month-old scallops (Figure 2). According to the results, progesterone was relatively higher in the ovaries than testes throughout the development, with an average of 1,048–2,333 pg/g in the ovaries and 382–1,345 pg/g in the testes (Figure 2A). Significant difference in the contents of testosterone between the ovaries and testes was observed in 3- to 10-month-old scallops, with the testes having a higher level of testosterone (Figure 2B). But a significant lower level of estradiol in the testes than the ovaries was found in 2- to 10-month-old scallops (Figure 2C). Smaller concentration variations were found for testosterone in the ovaries (171–337 pg/g) than testes (234–713 pg/g), and for estradiol in the testes (205–489 pg/g) than ovaries (298–1,109 pg/g) during the gonadal development. The estradiol/testosterone (E_2_/T) ratio was consistently higher in the ovaries than the testes in 2- to 10-month-old scallops (Figure 2D).

### Identification of *A. irradians FoxL2* and *Dmrt1L*

**FIGURE 3:**
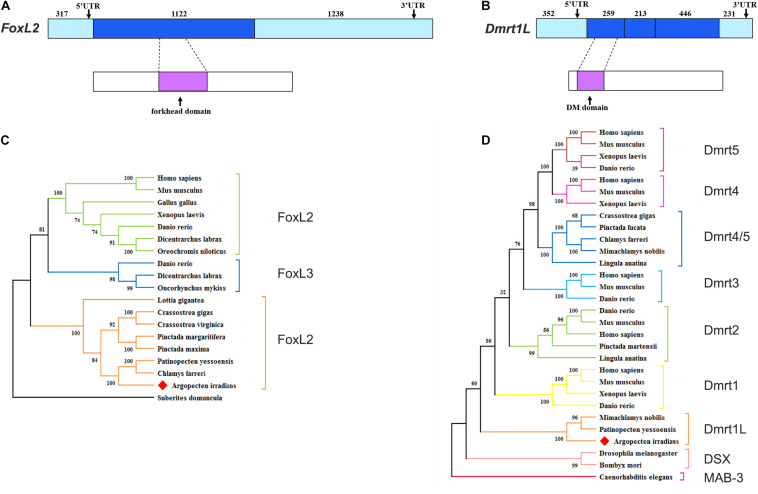
Structures and phylogenetic analyses of FoxL2 and Dmrt1L. **(A)** Gene and protein structures of FoxL2. **(B)** Gene and protein structures of Dmrt1L. The light blue box indicates the 5′UTR and 3′UTR. The blue box indicates the exon. The magenta box indicates the conserved functional domain. **(C**) Phylogenetic analysis of FoxL2. **(D)** Phylogenetic analysis of Dmrt proteins. The tree was constructed using the NJ method, and the numbers indicate the bootstrap percentage (1,000 replicates). Different branch colors denote different groups, and red diamonds indicate *A. irradians* FoxL2 and Dmrt1L. The registration numbers of the protein sequences used in the phylogenetic tree are shown in Supplementary Material 1, and the sequence alignments are shown in Supplementary Material 3.

FoxL2 and Dmrt1L were reported as key transcription factors regulating the differentiation of ovary and testis in the scallop *Patinopecten yessoensis* (Li et al., 2018). In present study, we identified both of them in the bay scallop by using the gonadal transcriptome data of *Argopecten irradians*. The full length of FoxL2 and Dmrt1L cDNA is 2,677 and 1,501 bp, encoding 373 and 305 amino acids, respectively (Supplementary Material 2). The molecular weight of FoxL2 and Dmrt1L proteins was predicted to be 42.47 and 33.67 kDa, respectively. FoxL2 has a highly conserved winged helix DNA binding domain called the forkhead domain (Figure 3A), and Dmrt1L possesses a conserved DM domain (Figure 3B). Phylogenetic analysis revealed that *A. irradians* FoxL2 grouped with FoxL2 from other mollusks, and then clustered with vertebrate FoxL2 and FoxL3 (Figure 3C). *A. irradians* Dmrt1L, together with its homologs from Yesso scallop *P. yessoensis* and noble scallop *M. nobilis*, form a single clade, instead of clustering with the well-characterized vertebrate Dmrts (e.g., Dmrt1, Dmrt2, Dmrt3) or Dmrts of other invertebrates (e.g., Dsx, Mab-3) (Figure 3D).

### The Sexually Dimorphic Expression of *FoxL2* and *Dmrt1L*

**FIGURE 4:**
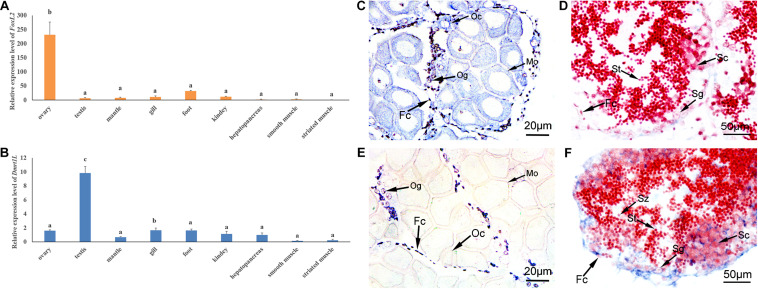
Spatial expression patterns of *FoxL2* and *Dmrt1L* in the adult tissues. **(A,B)** Relative expression level of *FoxL2* **(A)** and *Dmrt1L* **(B)** in nine adult tissues by RT-qPCR. The vertical bars represent the means ± S.E. (*N* = 4). One-way ANOVA followed by Duncan’s test was performed to compare the differences among tissues. Different letters indicate significant differences (*P* < 0.01). **(C,D)** Localization of *FoxL2* with an anti-sense probe in the ovary **(C)** and testis **(D)**. **(E,F)** Localization of *Dmrt1L* with an anti-sense probe in the ovary **(E)** and testis **(F)**. Positive signals with an antisense probe are indicated in blue. The red color was given by staining with neutral red as the background. Fc, follicle cell; Og, oogonium; Oc, oocyte; Sg, spermatogonium; Sc, spermatocyte; St, spermatid; Sz, spermatozoon.

In order to verify the feasibility of FoxL2 and Dmrt1L as sex markers in *A. irradians*, we first examined the expression of *FoxL2* and *Dmrt1L* in nine adult tissues, including ovary, testis, mantle, gill, foot, kidney, hepatopancreas, smooth muscle and striated muscle. According to RT-qPCR results, *FoxL2* was mainly expressed in the ovary, and the level was approximately 40-fold higher than that in the testis (Figure 4A). *Dmrt1L* could be detected in various tissues, but its expression in the testis was significantly higher than the other tissues (Figure 4B).

We further investigated the spatial expression of *FoxL2* and *Dmrt1L* in the ovary and testis by *in situ* hybridization. As can be seen, the signal of *FoxL2* transcripts was strong in the cytoplasm of oogonia, oocytes and follicle cells of the ovary (Figure 4C), and faint in the follicle cells of the testis (Figure 4D). The anti-sense probe of *Dmrt1L* could be detected in the ovary, but the signal exists only in the oogonia and follicle cells (Figure 4E). In the testis, *Dmrt1L* transcripts were located in various types of cells including the spermatogonia, spermatocytes and follicle cells (Figure 4F). No signal was detected in the ovary or testis with the sense probes (not shown). The dimorphic expression pattern of *FoxL2* and *Dmrt1L* in the gonads confirmed their feasibility as markers for sex identification in *A. irradians*.

### The Dynamic Changes of *FoxL2* and *Dmrt1L* During Gonadal Development

**FIGURE 5:**
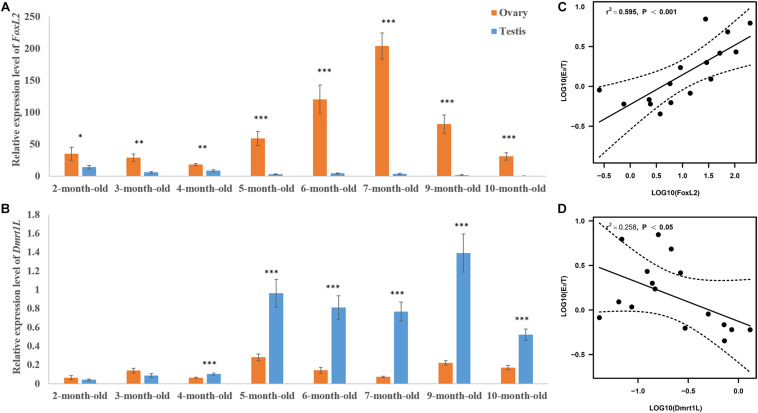
Expression patterns of *FoxL2* and *Dmrt1L* in the gonads and their correlation with E_2_/T ratio. Relative expression level of *FoxL2* **(A)** and *Dmrt1L* **(B)** in the ovaries and testes of scallops aged 2–10 months old. The vertical bars represent the means ± S.E. (*N* = 8). “^∗^” indicates *P* < 0.05; “^∗∗^” indicates *P* < 0.01; “^∗∗∗^” indicates *P* < 0.001. **(C)** The positive correlation between expression of *FoxL2* and E_2_/T ratio (*N* = 16). **(D)** The negative correlation between expression of *Dmrt1L* and E_2_/T ratio (*N* = 16). Dotted lines represent confidence interval of 95%.

We then tracked the dynamic changes of *FoxL2* and *Dmrt1L* expression during gonadal development. As shown in Figures 5A,B, both genes were detected in the ovaries and testes of 2- to 10-month-old scallops. The expression of *FoxL2* was higher in the ovaries than testes as we expected, and the difference was significant throughout the whole developmental process. In contrast, expression of *Dmrt1L* was similar between the ovaries and testes in 2- and 3-month-old juveniles, and sexual dimorphic expression of *Dmrt1L* did not occur until the scallops reached 4-month old. In addition, the difference of *FoxL2* and *Dmrt1L* expression between ovaries and testes was relatively small in 2- to 4-month-old individuals, and more obvious variation could be observed thereafter.

### The Relationship Between *FoxL2* and *Dmrt1L* Expression With *E*_2_/*T* Ratio

To illustrate the role of FoxL2 and Dmrt1L in gonadal development, we performed correlation analysis between the expression of *FoxL2* and *Dmrt1L* with E_2_/T ratio. According to the results, log10(E_2_/T) has a significantly positive correlation with *FoxL2* expression (*r* = 0.77, *P* < 0.001) (Figure 5C), and a significantly negative correlation with *Dmrt1L* expression (*r* = −0.5, *P* < 0.05) (Figure 5D).

### Summary of the Developmental Process of *A. irradians* Gonads

**FIGURE 6:**
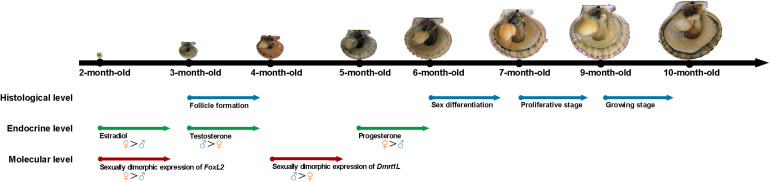
Schematic diagram of the gonadal development of *A. irradians* based on the histological, endocrine, and molecular levels. The arrows indicate the starting point of each event. Blue, green, and red colors indicate the histological, endocrine, and molecular levels, respectively.

According to the above results, we summarized the developmental process of *A. irradians* gonads in histological, endocrine and molecular levels (Figure 6). Significant higher levels of *FoxL2* expression and estradiol concentration are already found in the ovary of 2-month-old individuals. When scallops reach 3 months of age, the follicles start to form and there is a significant higher level of testosterone in the testis than ovary. Sexual dimorphic expression of *Dmrt1L* occurs from 4 months of age. In 5-month-old scallops, a higher level of progesterone can be found in the ovary than testis. One month later, sex is differentiated based on histological observation. Afterward, both the ovary and testis enter into gametogenesis.

## Discussion

FoxL2 and Dmrt are transcription factors that have been proposed to exert critical roles in sex determination, differentiation, and gametogenesis in various mollusks. In the present study, we identified single copy FoxL2 and Dmrt1L in *A. irradians*. As expected, *FoxL2* and *Dmrt1L* showed female- and male-biased expression in the gonad, respectively, similar to the expression patterns of *FoxL2* and *Dmrt* in other bivalves, such as the gonochoristic scallop *P. yessoensis* (Li et al., 2018; Nagasawa et al., 2019), the sequential hermaphrodites *C. gigas* (Zhang et al., 2014) and *P. margaritifera* (Teaniniuraitemoana et al., 2014, 2015). Spatial expression of *FoxL2* and *Dmrt1L* in the ovary and testis revealed that both genes were distributed in follicle cells and germ cells within the follicle, consistent with previous research in the gonochoristic scallop *C. farreri* (Liu et al., 2012) and *P. yessoensis* (Li et al., 2018), and the sequential hermaphrodite *C. gigas* (Naimi et al., 2009). Therefore, the role of FoxL2 and Dmrt1L in gonadal development could be conserved in gonochorism, sequential hermaphroditism and simultaneous hermaphroditism in mollusks. In vertebrates, FoxL2 and Dmrt1 have been demonstrated to antagonize each other in adult sexual maintenance (Zarkower, 2001; Ijiri et al., 2008; Smith et al., 2009; Uhlenhaut et al., 2009; Matson et al., 2011), and their expression pattern in the mangrove killifish *Kryptolebias marmoratus*, a simultaneous hermaphroditic species, is also similar to the gonochoristic fish species (Qu et al., 2020). It seems antagonism between FoxL2 and Dmrt member could be deeply conserved for sexual development in diverse taxa of metazoans.

Histological analysis is most widely applied for determining the onset of sex differentiation. But with the development of sex markers, timing of sex differentiation can be determined in a molecular way (Haugen et al., 2012; Ayers et al., 2013; Robledo et al., 2015; Tao et al., 2018). In this study, we found morphological sex differentiation occurred at 6 months of age in *A. irradians*. However, sexual dimorphic expression of *FoxL2* and *Dmrt1L* occurred at 2 and 4 months of age, respectively. It suggests that sex differentiation occurs earlier at the molecular level than at the histological level. Similarly, morphological sex differentiation occurred at 10 months of age in *P. yessoensis*, about 3 months later than the molecular sex differentiation (Li et al., 2018). These studies indicate that relative expression of *FoxL2* and *Dmrt1L* allows early identification of sex, and is a convenient and accurate way to assess timing of sex differentiation in scallops, and possibly in other mollusks as well. Interestingly, we found sexual dimorphic expression of *FoxL2* occurred earlier than *Dmrt1L*, suggesting that *FoxL2* may be the key driving factor of sex differentiation in *A. irradians*, and further research on the molecular mechanism underlying sex differentiation should be conducted in 2-month-old or even younger individuals.

In mollusks, controversy exists regarding the role of sex steroid hormones in gonadal development. Sex hormones displayed sex differences in some organisms. For example, higher level of testosterone and lower level of estradiol in the testes than ovaries have been reported in *C. farreri* (Liu et al., 2014), *Crassostrea angulata* (Ni et al., 2013), *Sinonovacula constricta* (Yan et al., 2010), *Marisa cornuarietis* (Janer et al., 2006), and *Ilyanassa obsoleta* (Sternberg et al., 2008). But in *Scrobicularia plana*, *Mya arenaria* and *Nucella lapillus*, no significant differences in steroid content was detected between genders (Santos et al., 2005; Gauthier-Clerc et al., 2006; Mouneyrac et al., 2008). In present study, we found significantly higher levels of testosterone and lower levels of estradiol in the testes than in the ovaries almost during the whole developmental process in *A. irradians*, suggesting the two hormones likely participate in sex differentiation and subsequent gametogenesis.

It is well known that in vertebrates, androgens can be transformed into estrogens by aromatase, which is encoded by Cyp19A1. Moreover, Cyp19A1 is a target of FoxL2, but it only exists in vertebrates (Batista et al., 2007; Markov et al., 2009; Callard et al., 2011). The presence of estradiol in a diversity of mollusks suggests there could be alternative genes encoding aromatase in these organisms. To examine whether scallop has the ability of androgen-to-estrogen conversion and if FoxL2 participates in regulating this process, we used E_2_/T to evaluate aromatase activity and analyzed its relationship with *FoxL2* expression. Interestingly, we observe a highly significantly positive correlation between E_2_/T ratio and *FoxL2* level, suggesting that in *A. irradians*, FoxL2 may target an alternative gene to vertebrate Cyp19A1, which encodes an enzyme that enables androgen-to-estrogen conversion. Previous reports on aromatase activity in *C. gigas* (Le Curieux-Belfond et al., 2001) and *Mytilus trossulus* (Hallmann et al., 2019), and our recent study in *P. yessoensis* (Zhang et al., 2020) also support this speculation. Therefore, we assume this could be the way FoxL2 drives sex differentiation and gametogenesis in mollusks.

## Conclusion

In summary, we investigated the gonadal developmental process of *A. irradians* at the morphological, endocrine and molecular levels. We found morphological sex differentiation occurred at 6 months of age, about 4 months later than sexual dimorphic expression of *FoxL2*. Moreover, the expression of *FoxL2* was significantly correlated with E_2_/T, suggesting FoxL2 may function by regulating the enzyme that converts androgen to estrogen. Further research on the molecule that links between FoxL2 and sex hormones would undoubtedly assist in unraveling the mechanism underpinning sex differentiation in mollusks.

## Data Availability Statement

The original contributions presented in the study are included in the article/Supplementary Material, further inquiries can be directed to the corresponding author/s.

## Ethics Statement

The animal study was reviewed and approved by the Ocean University of China.

## Author Contributions

LLZ and ZB developed the research questions and designed the experiment. HW, WL, and TL performed research. HW and WL completed the analyses and wrote the first draft of the manuscript. QX, YL, YS, LL, and LJZ aided in sample and data collection. LLZ and SW edited the manuscript. All authors contributed critically to the drafts and gave final approval for publication.

## Conflict of Interest

The authors declare that the research was conducted in the absence of any commercial or financial relationships that could be construed as a potential conflict of interest.

## Footnotes

1 http://smart.embl.de/

## Supplementary Material

Supplementary Material 1 The registration numbers of the protein sequences used in the phylogenetic tree.

Supplementary Material 2 The full length of FoxL2 and Dmrt1L cDNA sequences of *Argopecten irradians*.

Supplementary Material 3 The alignments of sequences used for the phylogenetic analyses.
