# Supplementary material for: Sexual Development of the Hermaphroditic Scallop Argopecten irradians Revealed by Morphological, Endocrine and Molecular Analysis
Source: Front Cell Dev Biol. 2021 Mar 16;9:646754. doi: 10.3389/fcell.2021.646754 (PMC8007870; doi:10.3389/fcell.2021.646754)
Supplement: Supplementary Material 2 — The full length of FoxL2 and Dmrt1L cDNA sequences of Argopecten irradians. [file Table_2.DOCX]

**Supplementary Material 2**

**The full length of FoxL2 and Dmrt1L cDNA sequences of *Argopecten irradians*.**

FoxL2

GGGATGTACATTTCTGTCGTATTAAGTGGAAATTGACAAGCTAGCACCCCCTATCCCAGTCACGTGACCGGTGAGGTGGAGTGAGCGGTATATATACAAACATTTTGATTAGTTTTCAATAACTTGTTGGTGTTGGTGTAGTTGACGCTGGATGCAGTTCTATTAGAGTGCAAACTGTTTTGTTTGTTCCTGTCCAACATAGTTTATTTATATATCATATAACAATAATTGTTTGGATAGATCTCTTATCAGAGCCCATAATATTTTTGAACATTTGGGAATATCAAGCAACACTCTGAAAGAAATCTTTCAACTCGATGGCATTGTCGTTCTACGAGTCAAAGATGGAAGAAAATTCTGATTTTATGGACATAAAGTTTCGACTCTTTCGTGGAGGAAAAAGACCGGAGGCTTTTACTGACACTATCGAAAATACAAATGAAACTACAAAAGCATGGAAACCGCATGTTGGAAGTGGTTTGTCAACACCACATTATAAGTACTCTTGTACCCAAAAAAATCCAGTTTCATACGGTGTAGCAGCGAGACTTCAAGGGAATCAACAATGTGATGACAAACTCTCTGAAACGGAAGACCTGAAACCAATTAAAGCAGACAACACCAAAATCAAGGAAGAGTCATCATTAATGACAATAGCCAAAGATTCTAAATGCAGGAAGTTTGAGGAAGACACTAATAAATACTCGGATCCGGATCAAAAACCTCCGTTTTCTTATGTAGCCTTAATTGCCATGGCAATCAAAGAATCAGGCGACAAGCGTTTGACTTTGAGCGGTATATATCAGTTTATCATCAGCAAGTTTCCTTATTATGAACGCAACAAGAAAGGATGGCAAAACAGCATCAGACATAACCTAAGCCTGAATGAGTGTTTCGTCAAGGTTCCCAGAGAAGGTGGCGGTGAACGGAAGGGTAACTTCTGGACCCTAGACCCTGCCTTCAACGACATGTTTGAAAAGGGAAATTATCGCCGACGCCGACGAATGCGGAGGCCATATCGCGCAGCTATATCTCTTCCTAAACCCCTTTTTGCCGACAACCATTGTGGACCTTATAACCAGTTTGCTCAATTAACAAAGCCCTATTTCTCACCGCCCCCTTATTCTCAGTACTCACAATACTCCCCTTGGGCCCTTACCCACAATGCCTCAGCACACCAGGGGATGGGCATGTCCCAGATCAGTAACTTTAACTCATGTACGCAGGCCCGTGTACCCCCTCCTGGCTCTACCTTAAGTTCCTGTGGGTACAATCCCCTACCCTCGGGGGTACAACTCTCTCCCAGTGCAGGTACAACCTACTCGCAACTCAATGACTACAACTCCGTGAGTCCCTCGGGACCCTTTCCATTCAGCCCCTACCGCCAGCAGGGAGAATCCTTCAACGCAGTCCACTACACGTACTGGGGAGAAAGATGATCAGATACAGACAGTTTGGAACGGACACATTGAAACATTTTGAATAATTGTTTCCTGAGCGAGGGATGTTTGAAATTTTAATAACTGAAACTGTTTCGGAAACAGAATTTCTACAAAGTGCTTCAGTTATTCTGAAGATTCCATGAAAACAGTTCTGCCTTTTGAAAACGTTTGGATTTTTGTGTGCAGGTGTTTATTGTTACGACATTGCTTTAGTGTTTACAAAAAATCAGACAATATCTGAAAATATAGGACGTTACTTTCCATGTTTCGAGACACGTCTGCTCTACATCACACAAGTAATACTGCAGTCATGGGAACTTTCCTCGATTATTTCAAATAAAACCGGGAAAGACTGAATAGTATGCCCTGAATCTGCGACCATACTGTAAGATTCCGTGGATTTACATAGTCATGTGAAACCTTGACCAGAAAACCAGACATTCTTCATTAACATACAATAAAATAAATAATTTGAGATGCTTTTTCAATTAAGTTGGACGATTGTCATTTTTCTATAGACATATTTGATATTTAATTTCATGTGCTGTTATTATATTATTAACTTAGACTATATACATCTGTAGCTATAAACTCTGCTATGTATAACTTTGTGTAACGCACCTTAGACACCCAAAGTAATTAGCACCTGTGGGGGATGGTCAGCTGGTATCAACCACACAACTAAATATATTTTCAGGTTACACAAAATCATATAAATCATATGTTTTATTGGACTTTTATTAAATGGTATTTAATTGCTTGGATTTTAAGTATTTTCGATTTGATGTTTGAAAATGTTTTAAAAGTCAGCCGATCAACATTAAAACCTCGCATGTTAAGGAGAAAATTTATGAAAATCGCCCACTTACTCAAAACTCCTAAGTGTATCTTGATATATTGTACAGGATGTTTAAGACGTGGGAGTTAATTTAAAACTGTTACCTGTAGGTGTTCTGCTTACCTGCTTAACACTTTCATGGTTTCAAACTGGCATGAAAGGGACAGAATAACGGTCGGTTTTCTTTATAACCATTAATTTTTAGAGACCCATTTGAGTGGTATATAGGGTTGGAATAAGCTAGATTCTATGTTATTATATCCACAGTCGAAAAAAACTTAAAAAACGCACATAAATTTTTAGAAAATATTTCGTTGGTAGCTTTGTACATTCTTAATTATTTCAAAATTACTTGTATACCATGTACATGTTAATAATTAATAAAAACAAAGAAGTA

Dmrt1L

ATAATGAGTACCTGATTGATGTAGAGTCATCTGGTGTGAGCCCCGACTTGCAGCTAGTAATGAGAAGGGAATGGGATCAAGTTTGAATACACAACGTTCAAAGACTTTTTATCTGTGACACTATTAACACTGGTAAATGTTTTAGCCTGGGCTGTTGCATCAAAGCCGGGCGCTCGTTTTTCAAACTCGGAGCTGTACACAAAAATGACGGCGAAAGCAACGTCTTCAGGGTGCAAGGTCATGGTTGACACTTTCCCACGAGTCAATCATGTCACGTGGTCACAGAGTGCACTATGCATCTCCAAGAAATACGCCTATACCAGAAGTTGTTGGACCAGGATCTTGAAAGCTTATGTCTACGAAGAAAGAAACAAAGCCCGCGGTGGGCCTATCTCCACTACGCTCCCCCAAGTGTTGTCGGTGCCGAAACCATGGCATCATAACCGTTTTGAAGGGCCACAAGCGGTTTTGTCAATGGAAAAATTGTACGTGTGATAATTGTCTTCTGTTGATGAAGCGCCAACAAAATTCTAAAGAACAAATTGCCCTGAGGCGTCTATGGAAACAAGAGGAGGATATGGGTCTGGTGGCGCCTACACCCGTTAGTACAGATTCCCTACAGATCCTCATTCATCGATATCCACACTACAACGTCGACAAATTGGGCGCCATATTGAAGTCATGTGACGGAGACACTCAGAAAGCCATAGAGAGGATTGACGCTGTCACAAACAGGGCCCTAGATGCCATTACGACCGAACCTAAGGCAGGAATGACACCTTCCTGCGCCGGGGATATGTGTGTCTCTCCAGGAAACACCGCCGGAATGGGATTTGTTGGTTTCCAGGGGTATAACTCTGGATTCCATACCCCTCTACCTCCGCCCCCCTCGGCCGTGAACCCCCTGTCCCCCCGGGCTAGTATGCAGATGTACCCGAGGACGCCCTATAATATGTACCCCTTCAGTCCCCCACGATTCTACCCCCCCACTCAGCAGGATATATCTTACAACGGGGACTTCCATGGTCCAACCCCACGATCTGGACATTCTGCTCAAAACTTCCAGACCTCCCGGAACGCATTTTATTCGGAATGCCAAAGTTCGGTAGAGTCGCTGATTGGATATCCCAAAGTTTCGGAAGCAGGCGTGTACCAGTCGCGTCCCTCAACTGCCAGCTCCGAGACGGAAGGCAACCTTGTTATCGATATGAAGGATGAGGAGGAGAGCGTTTGCGCGGAGGGACTTCTCACGAGCTACCCCACCCAGTGACGTCTTGGGATTTAACACAGATGAACTATTCATCTTTAAAAAGATTAGGATATTGATTTGGGTAATAACACAAGAATAGAATGCGCCTGTCTTATAGACTATATATAGTAAAGTGATTTTGTTGTAATTGATTCTCTTTTAAAAAATGTTATATGAAGTTTATTGTATATACGTTATTATTTCGATAGCTAAAGATAAATTACAAATGTACATATTAAAGAAAATTTCAAC
