# Supplementary material for: Sexual Development of the Hermaphroditic Scallop Argopecten irradians Revealed by Morphological, Endocrine and Molecular Analysis
Source: Front Cell Dev Biol. 2021 Mar 16;9:646754. doi: 10.3389/fcell.2021.646754 (PMC8007870; doi:10.3389/fcell.2021.646754)
Supplement: Supplementary Material 3 — The alignments of sequences used for the phylogenetic analyses. [file Table_3.DOCX]

**Supplementary Material 3**

**The alignments of sequences used for the phylogenetic analyses.**


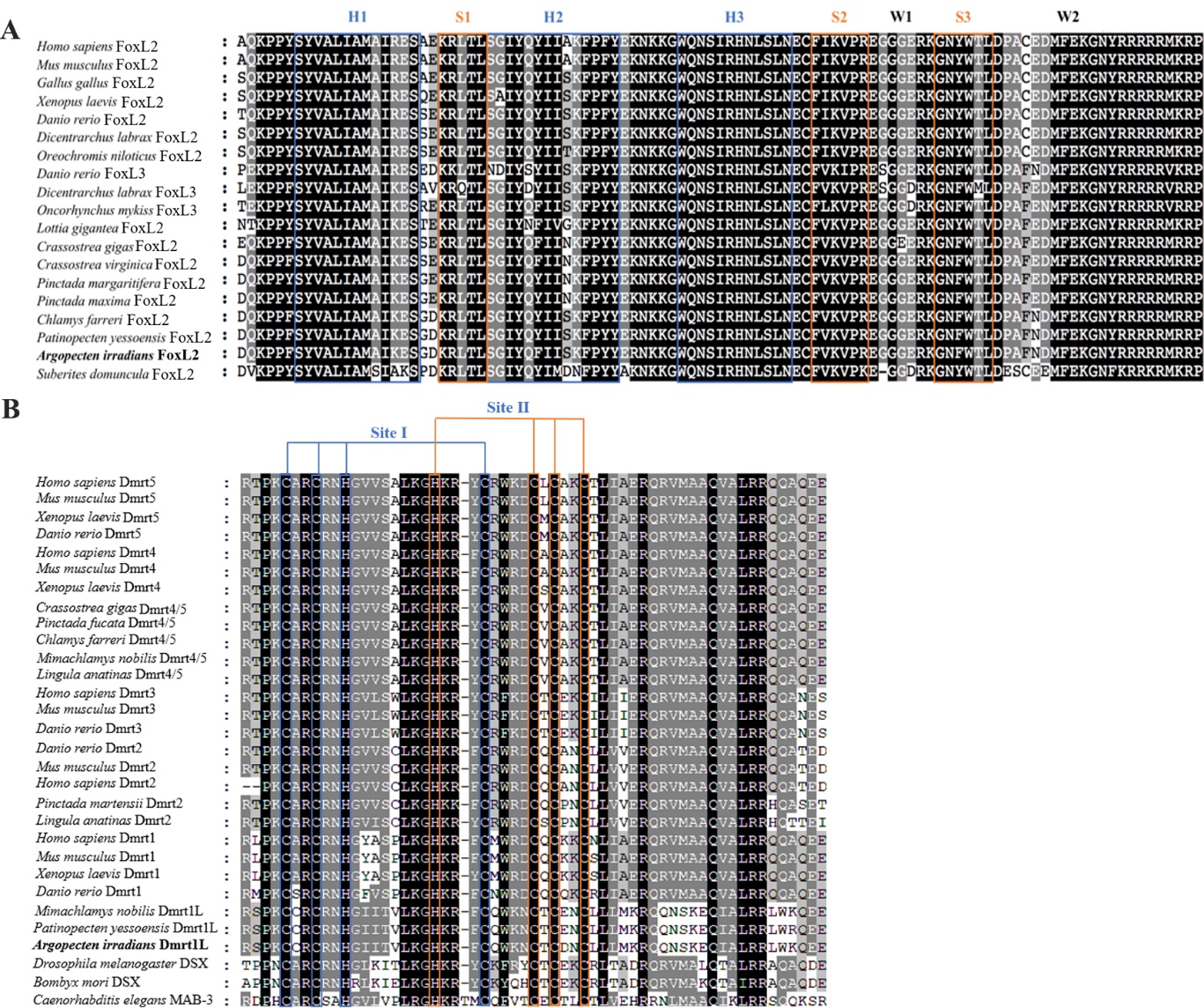


(A) Alignment of the forkhead domains of FoxL2 homologs. The three helices (H1~ H3; blue boxes), β-strands (orange boxes) and two wings (W1, W2) structuring the forkhead domain are indicated. (B) Alignment of the DM domains of Dmrt proteins. The zinc module consisting of intertwined CCHC and HCCC Zn^2+^-binding sites is shown with blue (Site I) and orange (Site II) boxes.
